# Impact of Acute Lymphoblastic Leukemia Treatment on Left Ventricular Function Assessed in 2D and 3D Speckle Tracing Echocardiography—Preliminary Results

**DOI:** 10.3390/jcm14248682

**Published:** 2025-12-08

**Authors:** Julia Haponiuk-Skwarlińska, Halszka Kamińska, Katarzyna Albrecht, Paweł Łaguna, Bożena Werner

**Affiliations:** 1 Department of Pediatric Cardiology and General Pediatrics, Medical University of Warsaw, 02-091 Warsaw, Poland; 2 Department of Pediatrics, Oncology, Hematology and Transplantology, Medical University of Warsaw, 02-091 Warsaw, Poland

**Keywords:** left ventricular echocardiography, speckle tracing, cardiotoxicity, pediatric cardio-oncology, acute lymphoblastic leukemia

## Abstract

**Background/Objectives**: Acute lymphoblastic leukemia (ALL) is the most common childhood malignancy with promising survival. ALL treatment involves the use of cardiotoxic anthracyclines. The data for children on new methods of echocardiographic detection of early-onset anthracycline-related left ventricle dysfunction is lacking. **Methods**: Consecutive children with ALL were prospectively enrolled. The echocardiography was performed after ALL diagnosis and before initiation of anthracyclines (first assessment) and after induction and intensification therapy completion (second assessment). The left ventricle echocardiographic assessment involved conventional two-dimensional (2D) echocardiography, 2D speckle tracing, and three-dimensional (3D) echocardiography with offline analysis for 3D speckle tracing. **Results**: The preliminary group of 32 children presented with mean time between the first and second assessment of 7.3 ± 1.5 months (min.5.3–max.11.4). All children were treated by the same treatment protocol and received doxorubicin and daunorubicin. The mean cumulative equivalent anthracycline dose was 165.6 ± 54.0 mg/m^2^. Statistically significant differences between the first and the second echocardiography were observed in LV-GLS-2D −24.6 ± 3.3% vs. −21.0 ± 3.3%; *p* < 0.001, LVEF-3D 59.7 ± 7.3% vs. 55.1 ± 3.0%; *p* = 0.010, LV-GLS-3D −23.3 ± 5.3% vs. −20.4 ± 2.8%; *p* = 0.031 and LV-GCS-3D −26.3 ± 5.9% vs. −21.9 ± 3.2%; *p* = 0.017. The differences in LVEF-2D and LV-GRS-3D were not statistically significant. The decrease of >15% from the first assessment was observed in 9 (28%) in LV-GLS-2D, 8 (25%) in LV-GLS-3D, 11 (34%) in LV-GCS-3D and only in 4 (13%) patients in LV-GRS-3D. **Conclusions**: Two-dimensional and three-dimensional speckle tracing and 3D-LVEF may be sensitive indicators of subclinical left ventricular function impairment in children treated for ALL with anthracyclines. However, this is a preliminary analysis of the planned cohort; our results should be interpreted with caution.

## 1. Introduction

The predominant malignancy in the pediatric population is acute lymphoblastic leukemia (ALL) [1]. Although the survival rate is promising, ALL treatment involves the use of cardiotoxic anthracyclines [2]. Childhood cancer survivors who have received anthracycline therapy are at increased risk of developing symptomatic heart failure, with reported incidences reaching up to 10.6% at 40 years post-diagnosis [3]. This elevated risk may be attributed to left ventricular systolic dysfunction [4] and, in some cases, valvular heart disease [5]. The cardiotoxicity related to anthracycline use can be detected in speckle tracing echocardiography among adult childhood cancer survivors and adult oncological patients [6,7]. Currently, the European Society of Cardiology (ESC) recommends performing echocardiography in adults treated with anthracyclines before and 12 months after treatment, and in long-term follow-up [8,9]. The anthracyclines in ALL treatment are used during the induction and intensification phases (usually the first 6 months of a total of around 24 months). Data on echocardiographic detection of early-onset anthracycline-related left ventricular dysfunction in children are lacking.

Based on cardiac function assessment studies in adult populations, we hypothesize that the newest echocardiographic methods may enable a comprehensive evaluation of the impact of ALL treatment on children’s cardiac function. Next, 2D and 3D strain echocardiography of the left ventricle (LV) are reported as more sensitive methods for systolic function assessment and can potentially detect subclinical cardiac function impairment from cardiotoxicity of ALL treatment in children.

In this report, we present preliminary results of a comprehensive left ventricular function assessment using 2D and 3D echocardiography, including speckle tracking, in children treated for acute lymphoblastic leukemia before initiation of anthracycline treatment and after the induction and intensification phases of the therapy.

## 2. Materials and Methods

We present a preliminary report on the impact of anthracycline treatment on left ventricular function, assessed by 2D and 3D echocardiography, in children currently treated for ALL.

In this paper, a preliminary report on echocardiographic results from the prospective observational study, “Assessment of chemotherapy-induced cardiac dysfunction and exercise capacity in pediatric patients treated for acute lymphoblastic leukemia,” funded by the Medical University, is presented. The study was performed at the pediatric tertiary center—Pediatrics and Pediatric Cardiology Department in cooperation with the department of Pediatrics, Oncology, Hematology, and Transplantology. The patient enrollment time for the preliminary study was January 2023–March 2024. The study participants were consecutive children diagnosed and treated for ALL, the first to be recruited according to the inclusion and exclusion criteria (Table 1) after written informed consent. To solely analyze the impact of anthracyclines on cardiac function, children treated with other cardiotoxic treatment methods (radiotherapy or hematopoietic cell transplantation) were excluded from the analysis.

The sample size group required for the study was 60 participants, determined statistically (power of 80% and a level of significance of 5%). The preliminary group of 32 participants showed statistically significant changes in echocardiographic parameters that we believe may be clinically important.

The primary outcome of interest was detection of contractile left ventricle function impairment—2D and 3D strain echocardiography results below the reported normal values for children and ejection fraction (EF) 55% or less [8,9].

### 2.1. Data Collection

For the preliminary study, baseline anthropometric, demographic, and clinical data were collected from medical records, based on patients’ treatment documentation, ensuring consistency and measurement standards. No more than 9% of retrospective medical data was missing for each variable. Anthracyclines included doxorubicin and daunorubicin. The 0.6 cardiovascular toxicity dose ratio was used for the daunorubicin equivalence and cumulative anthracycline dose calculation [8].

### 2.2. Echocardiography

2D- and 3D echocardiography was performed after diagnosis and before initiation of anthracyclines (first assessment, 10–14 days after diagnosis) and after completion of the induction and intensification therapy phases (second assessment, around the first 6 months after diagnosis). All studies were performed by two experienced echocardiographers on an EPIQ CVx 5.0 ultrasound machine (Philips Medical System, Bothell, WA, USA) and were obtained from at least two cardiac cycles. Standard echocardiographic parameters were obtained according to current recommendations [10].

**Figure 1:**
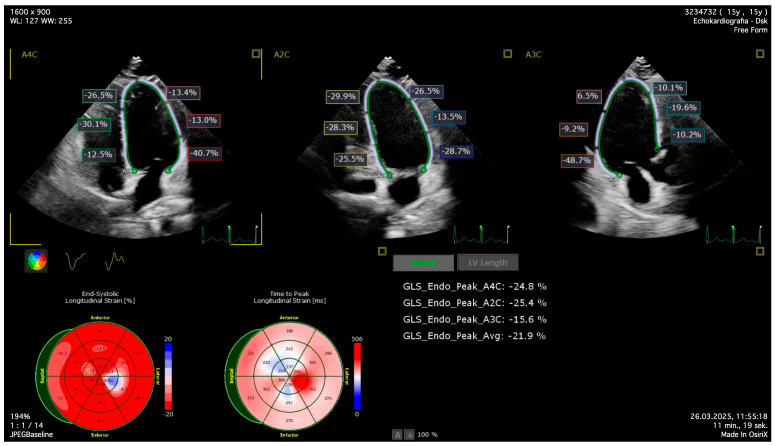
Figure to show LV-GLS-2D assessment.

**Figure 2:**
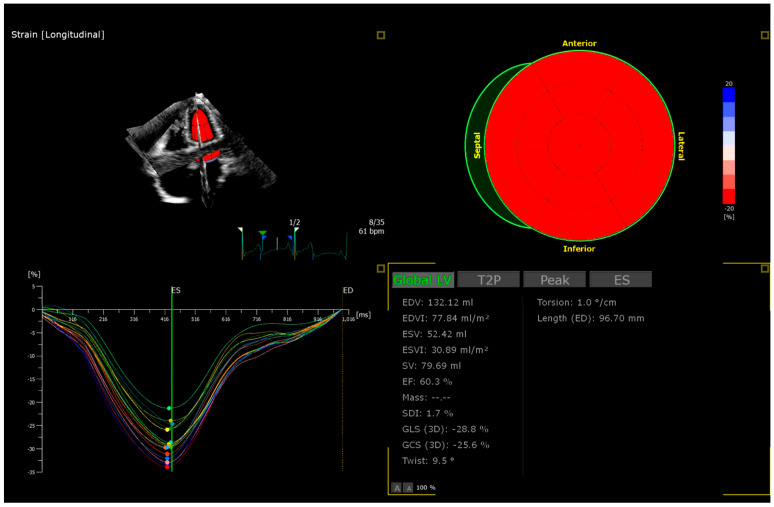
Figure to show 3D echocardiography assessment—LV-GLS-3D.

**Figure 3:**
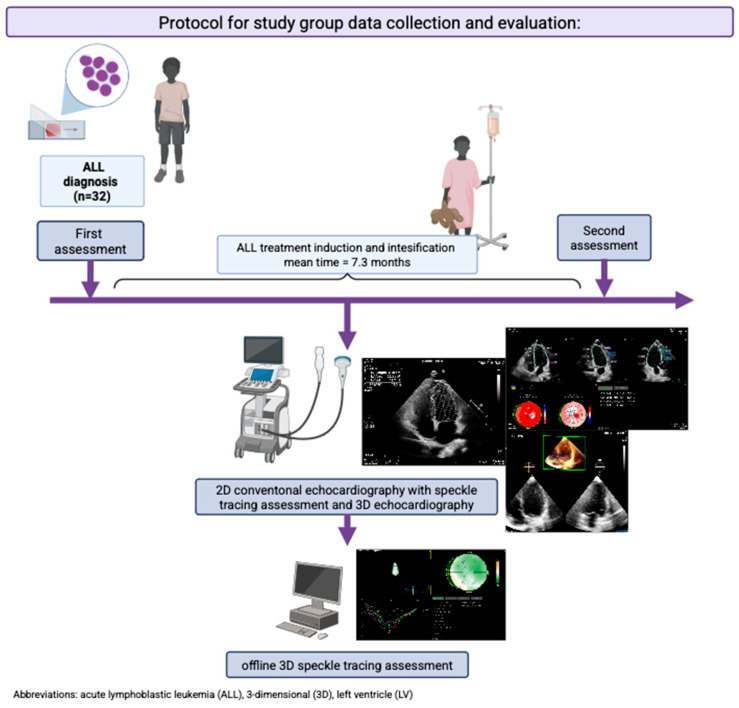
Figure to show protocol for study group data collection and evaluation. (Created in Biorender. Julia Haponiuk-Skwarlińska. (2025) https://BioRender.com/, created by Biorender.com).

The echocardiography involved 2D Simpson biplane left ventricle (LV) ejection fraction (LVEF-2D) and 2D Global Longitudinal Strain (LV-GLS-2D) in QLAB 2D strain software (Philips Medical System, Bothell, WA, USA) (Figure 1) and 3D assessment. GLS was measured from the apical two-, three-, and four-chamber views using a semiautomated assessment. Three-dimensional echocardiographic records were analyzed offline with the use of 4D LV-Analysis software (Philips Medical System, USA), assessing left ventricle ejection fraction (LVEF-3D), global longitudinal (LV-GLS-3D), global circumferential (LV-GCS-3D), and radial strain (LV-GRS-3D) (Figure 2). After automatic calculation of the records using the software, the operator manually adjusted endocardial borders if needed—no more than 10% of records were adjusted manually.

The echocardiographers were blinded to the imaging time point (pre vs. post) for the offline analysis. Images were anonymized before analysis and presented in a randomized order. Basic demographic information required for guideline-directed measurements (e.g., age, sex) was available, but no treatment or outcome data were accessible.

Interobserver and intraobserver reliability were assessed using a *t*-test and a correlation with 95% confidence intervals (Table 2).

The protocol for study group data collection and evaluation is presented in Figure 3.

This study was conducted according to the guidelines of the Declaration of Helsinki, and all procedures involving patients’ data were approved by the University Ethic Committee (Decision of Approval: KB/13/2023 from 16 January 2023).

### 2.3. Statistical Analysis

Statistical analysis was performed using the IBM^®^SPSS^®^, version 30.0.0. All the obtained data were checked for normality with the Shapiro–Wilk test. The variables were presented as mean (standard deviation) or as median with interquartile range. Comparisons between pre- and post-treatment echocardiographic measurements were performed using paired statistical tests, depending on data distribution (paired *t*-test for normally distributed variables; Wilcoxon signed-rank test for non-normal data).

A two-tailed α level of 0.05 was applied for all hypothesis testing. Missing data were handled using complete-case analysis; participants with unavailable paired measurements for a given parameter were excluded from that specific analysis only.

## 3. Results

This preliminary prospective study report is based on data from 32 children: 14 (43%) males and 18 (56%) females, diagnosed with ALL. The mean time between the first and second assessments was 7.3 (1.5) months (min. 5.3–max. 11.4). All children were treated according to the same protocol and received doxorubicin and daunorubicin. The mean cumulative equivalent anthracycline dose was 165.6 (54.0) mg/m^2^; five children received > 200 mg/m^2^ dosage for body surface area [5]. Detailed study group characteristics are presented in Table 3.

**Figure 4:**
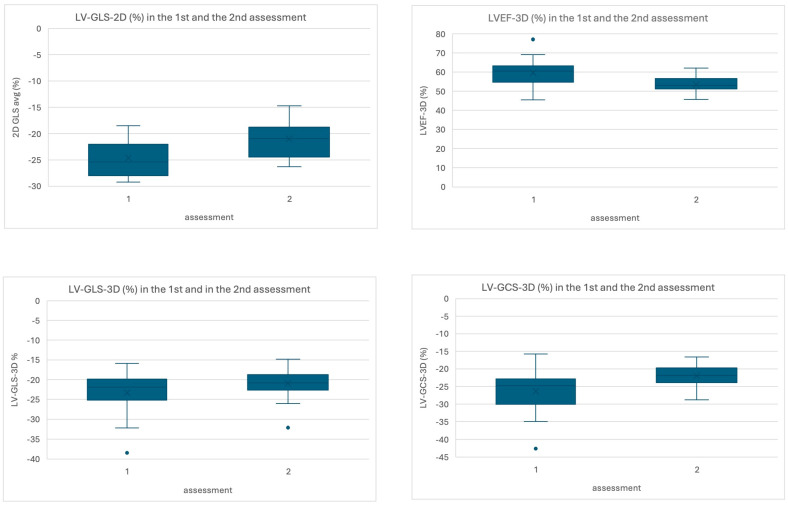
Figure to show mean results with error bars from the first and the second assessment of the LV-GLS-2D, LVEF-3D, LV-GLS-3D, and LV-GCS-3D.

Statistically significant differences between the first and the second echocardiography were observed in LV-GLS-2D −24.6 ± 3.3% vs. −21.0 ± 3.3%; *p* < 0.001, LVEF-3D 59.7 ± 7.3% vs. 55.1 ± 3.0%; *p* = 0.010, LV-GLS-3D −23.3 ± 5.3% vs. −20.4 ± 2.8%; *p* = 0.031 and LV-GCS-3D −26.3 ± 5.9% vs. −21.9 ± 3.2%; *p* = 0.017 (Table 4, Figure 4). The differences in LVEF-2D and LV-GRS-3D were not statistically significant. The decrease of >15% from the first assessment was observed in 9 (28%) in LV-GLS-2D, 8 (25%) in LV-GLS-3D, 11 (34%) in LV-GCS-3D and only in 4 (13%) patients in LV-GRS-3D (Supplementary Materials, Table S1).

Study group cardiac biomarkers levels at the second assessment were 2nd assessment 72.5 (50.8; 112.8) NT-proBNP (pg/mL) and 6.4 (0.0; 24.5) Troponin I/T (ng/mL). The time between the induction and intensification treatment completion and biomarker level assessment was up to 7 days from the completion of the intensification therapy.

## 4. Discussion

Our analysis suggests the possible occurrence of the early LV systolic impairment observed as a statistically significant decrease in the newest, sensitive contractility parameters including 2D and 3D LVGLS, 3D-LV-GCS and 3D-LVEF among children right after the completion of the anthracyclines therapy in ALL treatment. However, we would like to highligh that this is a preliminary analysis of a prospectively collected cohort and it must be interpreted with caution as final observations may differ from those reported here. 

Despite the decrease in strain values, all the observed values were within reported literature norms [11,12]. ALL survivors are at high risk of developing symptomatic HF during long-term follow-up. Anthracycline use within ALL therapy has been associated with abnormalities in LV function detected in conventional 2D echocardiography [13]. Since 2022, ESC advises LVEF assessment preferably with 3D echocardiography and LV-2D-GLS assessment to identify systolic impairment in adult patients both before and after anthracycline therapy (within the first 12 months after treatment completion) [8]. In survivor studies, it remains uncertain whether LV dysfunction is present early after therapy or evolves progressively over time. As echocardiographic monitoring during treatment was not routinely performed in earlier decades, comprehensive longitudinal echocardiographic data are limited. In our study, children with ALL were assessed earlier, right after completion of the induction and intensification therapy phases, suggesting another time point for echocardiographic assessment. Our results highlight the possible occurrence of early LV systolic dysfunction, which resembles the abnormalities observed in long-term follow-up (for example, 26% after a median of 18 years from diagnosis in González-Manzanares R) [14].

In addition, in adult patients, GLS values after initiation of potentially cardiotoxic chemotherapy with anthracyclines showed good prognostic performance for subsequent LV dysfunction defined by an LVEF decline [15]. Therefore, our results may further support the hypothesis for pediatric populations, but close echocardiographic follow-up must be performed.

Next, a proposed cut-off for patient assessment of potential LV function deterioration is a 15% reduction in GLS from baseline for patients with ALL [8]. In total, 25–34% of participants presented with a 15% reduction in strain values. Further analysis of the strain-reduction subgroup can assess the applicability of the cut-off level to the pediatric population.

Based on our results, we observed significant differences in both 2D and 3D speckle tracing values in children before and after the ALL intensive treatment phase. Few reports suggest that 2D-LV-GLS assessment in children undergoing ALL treatment is a more sensitive method than conventional 2D echocardiography for detecting early cardiotoxicity [16,17].

The Australian and New Zealand Delphi Consensus developed the first Cardio-Oncology Recommendations for Pediatric Oncology Patients, based on adult guidelines. The document recommends 3D echocardiographic measurements of LVEF and GLS as the optimal methods for LV assessment during the initial cardio-oncology consultation in children from high-risk groups (receiving ≥ 250 mg/m^2^ doxorubicin equivalent) [18]. The guidelines, however, lack specific follow-up time frames for children receiving only anthracycline therapy, as well as an approach to specific subgroups of patients (i.e., children with ALL). Adult ESC cardio-oncology guidelines from 2022 recommend extending periodic echocardiographic monitoring in long-term survivors of childhood cancer. The frequency of assessments should be based on cardiotoxicity risk, including exposure to anthracycline and radiotherapy [8]. Three-dimensional echocardiography is considered to have high accuracy for ventricular function assessment in pediatric patients [19], and it has been evaluated in adults receiving chemotherapy as well as in childhood cancer survivors [6,7]. To the best of the authors’ knowledge, no data on 3D LV speckle tracking assessment and its utility in children treated for ALL had been published before.

Our preliminary report focuses on echocardiographic findings; however, we have also observed changes in cardiac biomarker levels in the study group. The NT-proBNP elevation in children diagnosed with ALL before the treatment has been reported with leukemic infiltration and pressure overload as the main potential mechanisms [20,21]. After the intensification phase of ALL treatment, the study group presented with normal NT-proBNP values. Although the mean troponin I/T concentration is higher in the second assessment, the analysis, including the statistical assessment, is beyond the scope of this manuscript. Further, cardiac biomarker analysis and the assessment of possible correlations with 2D and 3D echocardiography are needed.

Early detection of subclinical cardiac dysfunction with highly accurate echocardiographic methods may enhance implementation of cardioprotective precautions and treatment. The results of our study highlight the need for major efforts on the development of adjusted, pediatric follow-up guidelines after cardiotoxic oncological treatment, as well as further improvement of sensitive echocardiographic methods of pediatric contractile function assessment.

### Limitations of the Study

The presented report is based on a preliminary study group for the prospective observation of the study. The small study sample did not enable us to reach statistical significance to analyze possible associations between echocardiographic results and cumulative anthracycline dose or cardiac biomarker levels within the study group. Our observations should be confirmed in a larger study group with yet another (shorter and longer) follow-up observation. What is more, we do not report indicative clinical endpoints of heart failure. Our cohort was homogeneous in ethnicity (Caucasian). Therefore, our results may not be applicable to other races, and further studies are warranted. Our current results should not be subject to selection and reporting bias.

## 5. Conclusions

Two-dimensional and three-dimensional speckle tracing and 3D-LVEF may be sensitive indicators of subclinical left ventricular function impairment in children treated for ALL with anthracyclines.

## Figures and Tables

**Table 1:** Inclusion and exclusion criteria for the study group.

Inclusion criteria
- age 0–18 years, - newly diagnosed acute lymphoblastic leukemia (ALL) based on bone marrow cytology (taken by fine-needle aspiration biopsy from one of the spines of the hip plate or tibia, according to the FAB classification of bone marrow smears stained by the May–Grünwald and Giemsa method), - qualification for treatment of acute lymphoblastic leukemia/lymphoblastic lymphoma with anthracycline chemotherapy, - written informed consent signed by parents and patient ≥ 12 years of age,
Exclusion criteria
- a history of cancer/ALL and treatment of cancer/ALL with chemotherapy, especially with anthracyclines, - acute infection, - platelet count < 20 G/L, - severe conditions: protein loss syndrome, peritoneal, pericardial, or pleural effusion, arrhythmia, metabolic disorders, - after initial chemotherapy that is common to all patients treated for ALL on the basis of response to treatment, qualification for a very high-risk group, and planned inclusion of radiotherapy or hematopoietic cell transplantation, - previously diagnosed chronic cardiovascular disease, i.e., cardiomyopathy, heart failure, congenital heart defects, arrhythmias. - significant chronic comorbidities involving the endocrine, neurological, and digestive systems, as well as the respiratory tract, and kidney diseases, - lack of consent from parent and/or patient > 12 years of age,

**Table 2:** Intra- and interobserver reliability for LV-GLS-2D, LVEF 3D., indicating *p* value from *t*-test and correlation coefficient (R) with 95% confidence interval.

	Interobserver		Intraobserver	
Parameter	*p* Value	R, 95% Confidence Interval	*p* Value	R, 95% Confidence Interval
LV-GLS-2D average	0.91	0.965, 0.835–0.978	0.95	0.996, 0.992–0.998
LVEF-3D	0.92	0.932, 0.732–0.984	0.94	0.986, 0.920–0.997
LV-GLS-3D	0.92	0.934, 0.738–0.985	0.97	0.986, 0.923–0.998
LV-GCS-3D	0.92	0.982, 0.925–0.996	0.95	0.981, 0.895–0.997
LV-GRS-3D	0.93	0.978, 0.906–0.995	0.93	0.976, 0.864–0.996

Abbreviations: LV-GLS-2D—two-dimensional global longitudinal strain of the left ventricle; LVEF-3D—three-dimensional left ventricle ejection fraction; LV-GLS-3D—three-dimensional left ventricle global longitudinal strain; LV-GCS-3D—three-dimensional left ventricle global circumferential strain; LV-GRS-3D—three-dimensional left ventricle global radial strain.

**Table 3:** The study group characteristics.

*Preliminary Study Group Characteristics* (*n = 32*)	
age at diagnosis (years old)	5.98 ± 3.7 (min. 1.6, max. 16.0)
sex n (%)	14 (43%) males, 18 (56%) females
Ethnicity n (%)	Caucasian n = 32 (100%)
diagnosis n (%)	31 (96%) ALL type C, 1 (4%) ALL type T
white blood cells at diagnosis	18.2 × 10^3^ (5.7 × 10^3^; 33.3 × 10^3^)
percentage of blasts detected in manual count (%)	85.2 ± 12.5
treatment protocol n (%)	AIEOP BMF 2017 n = 32 (100%)
initial treatment response n (%)	M1 n = 29 (90%) M2 n = 2 (6%) M3 n = 1 (4%)
BSA at diagnosis (m^2^)	0.87 ± 0.36
BMI at diagnosis (kg/m^2^)	15.9 ± 2.2
BMI percentile at diagnosis	37.9 ± 24.9
type of anthracycline used n (%)	Doxorubicin n = 32 (100%) Daunorubicin n = 32 (100%)
time from the first anthracycline dose (months)	7.3 ± 1.5 min 5.3–max 11.4
cumulative equivalent anthracycline dose (mg/m^2^)	165.6 ± 54.0 min. 97.2–max. 293.7
cumulative equivalent anthracycline dose > 200 mg/m^2^ n (%)	n = 5 (16%)
NT-proBNP (pg/mL)	
1st assessment	383.5 (116.0; 926.0)
Troponin I/T (ng/mL)	
1st assessment	0.0 * (0.0; 0.0)

Abbreviations: BSA—body surface area; BMI—body mass index. * laboratory threshold for troponin detection is >1.3 ng/mL.

**Table 4:** 2D and 3D speckle tracing results in children treated for ALL.

*Study Group—Children with ALL n = 32*					
Variable	First Assessment—at the Time of Diagnosis	Second Assessment—After Induction and Intensification Phase of Therapy Completion	*p* Value	Δ Difference (95% CI Lower/Upper)	Effect Size
2D Echocardiography					
LV volume (A4Cd) (mL)	52.0 ± 20.9	58.7 ± 24.1	0.13 ^t^	−4.9 (−11.3/1.5)	−0.329 ^cd^
LV volume (A2Cd) (mL)	44.4 ± 17.1	50.9 ± 20.2	0.21 ^t^	−4.7 (−12.3/2.9)	−0.267 ^cd^
EDV (BP) (mL)	49.0 ± 18.0	54.6 ± 21.4	0.35 ^t^	−3.2 (−10.0/3.7)	−0.206 ^cd^
LVEF-2D (Simpson Biplane) (%)	56.8 ± 8.4	55.4 ± 4.8	0.45 ^t^	3.1 (−0.8/7.0)	−0.329 ^cd^
LV-GLS-2D (%)	−24.6 ± 3.3	−21.0 ± 3.3	<0.001 ^t^*	−3.5 (−5.1/−1.8)	−0.865 ^cd^
3D Echocardiography					
EDV-3D (mL)	52.0 ± 23.1	55.6 ± 22.8	0.55 ^t^	−2.2 (−9.5/5.2)	−0.134 ^cd^
EDVI-3D (mL/m^2^)	58.8 (52.7; 64.9)	56.5 (47.3; 64.8)	0.31 ^w^	4.5 (−4.6/13.6)	0.233 ^wr^
ESV-3D (mL)	20.4 ± 8.5	25.8 ± 10.5	0.13 ^t^	−4.0 (−9.3/1.3)	−0.337 ^cd^
ESVI-3D (mL/m^2^)	23.2 ± 5.6	27.7 ± 7.0	0.02 ^t^*	−4.8 (−7.4/−2.3)	−0.883 ^cd^
LVEF-3D (%)	59.7 ± 7.3	55.1 ± 3.1	0.01 ^t^*	5.5 (1.8/9.2)	0.656 ^cd^
Length (ED) 3D (mm)	64.5 ± 8.9	66.2 ± 8.3	0.23 ^t^	−1.2 (−3.3/0.8)	−0.684 ^cd^
LV-GLS-3D (%)	−23.3 ± 5.3	−20.4 ± 2.8	0.03 ^t^*	−2.1 (−4.9/0.7)	−0.756 ^cd^
LV-GCS-3D (%)	−26.3 ± 5.9	−21.9 ± 3.2	0.02 ^t^*	−3.8 (−6.8/−0.8)	−0.555 ^cd^
LV-GRS-3D (%)	35.4 ± 20.0	35.9 ± 3.6	0.45 ^t^	2.4 (−4.1/8.9)	−0.165 ^cd^

^w^—Wilcoxon rank sum test; ^t^—Student’s *t*-Test; ^cd^—Cohen’s d; ^wr^—Wilxocon’s r; * is for statistically significant values. Abbreviations: ALL—acute lymphoblastic leukemia; 2D—two-dimensional; LV—left ventricle; A4Cd—apical four-chamber diastolic; A2Cd—apical two-chamber diastolic; EDV—end diastolic volume; LVEF-2D—two-dimensional left ventricle ejection fraction; LV-GLS-2D—two-dimensional global longitudinal strain of the left ventricle; 3D—three-dimensional; EDVI—end diastolic volume index; ESV—end systolic volume; ESVI—end systolic volume index; LVEF-3D—three-dimensional left ventricle ejection fraction; ED—end diastolic; LV-GLS-3D; three-dimensional left ventricle global longitudinal strain; LV-GCS-3D—three-dimensional left ventricle global circumferential strain; LV-GRS-3D—three-dimensional left ventricle global radial strain.

## Data Availability

Data available upon request.

## Supplementary Materials

The following supporting information can be downloaded at https://www.mdpi.com/article/10.3390/jcm14248682/s1. Table S1. Table to show the general incidence of lower 2D and 3D LVEF and >15% lower 2D and 3D strain values in the 2nd assessment of the preliminary study group.

## Abbreviations

The following abbreviations are used in this manuscript:

ALL	acute lymphoblastic leukemia,
2D	two-dimensional,
LV	left ventricle,
BSA	body surface area,
BMI	body mass index
A4Cd	apical four-chamber diastolic,
A2Cd	apical two-chamber diastolic,
EDV	end diastolic volume,
LVEF-2D	two-dimensional left ventricle ejection fraction,
LV-GLS-2D	two-dimensional global longitudinal strain of the left ventricle,
3D	three-dimensional,
EDVI	end diastolic volume index,
ESV	end systolic volume,
ESVI	end systolic volume index,
LVEF-3D	three-dimensional left ventricle ejection fraction,
ED	end diastolic,
LV-GLS-3D	three-dimensional left ventricle global longitudinal strain,
LV-GCS-3D	three-dimensional left ventricle global circumferential strain,
LV-GRS-3D	three-dimensional left ventricle global radial strain.
