# Supplementary material for: Impact of Acute Lymphoblastic Leukemia Treatment on Left Ventricular Function Assessed in 2D and 3D Speckle Tracing Echocardiography—Preliminary Results
Source: J Clin Med. 2025 Dec 8;14(24):8682. doi: 10.3390/jcm14248682 (PMC12734355; doi:10.3390/jcm14248682)
Supplement: Supplementary file 1 [file jcm-14-08682-s001.zip › jcm-3997963-supplementary.pdf]

Supplementary Materials

Table S1. Table to show the general incidence of lower 2D and 3D LVEF and >15% lower 2D and 3D strain values in the 2nd assessment of the preliminary study group.

n	LVEF-2D – lower in the 2nd assessment	LVEF-3D - lower in the 2nd assessment	LV-GLS-2D - >15% lower in the 2nd assessment	LV-GLS-3D - >15% lower in the 2nd assessment	LV-GCS-3D- >15% lower in the 2nd assessment	LV-GRS-3D - >15% lower in the 2nd assessment
1	1	1	0	0	0	0
2	1	1	0	0	1	0
3	1	1	1	1	0	0
4	1	1	0	0	0	1
5	1	1	0	0	0	1
6	1	1	0	0	0	1
7	0	1	0	0	0	0
8	1	0	0	0	0	0
9	1	1	0	1	0	0
10	1	0	0	0	0	0
11	0	1	1	1	1	0
12	0	1	1	0	1	0
13	1	1	0	0	1	0
14	1	1	0	1	0	0
15	0	1	0	1	0	0
16	0	1	0	0	0	0
17	1	1	1	1	1	0
18	0	1	1	0	1	1
19	0	0	0	0	0	0
20	0	1	0	0	1	0
21	1	1	0	0	1	0
22	1	1	1	1	1	0
23	1	1	0	1	0	0
24	1	1	1	0	0	0
25	1	1	1	0	1	0
26	1	1	0	0	0	0
27	0	1	0	0	0	0
28	0	1	0	0	0	0
29	0	1	1	0	0	0
30	0	0	0	0	0	0
31	1	1	0	0	1	0
32	0	0	0	0	0	0
	1 – yes, 0 - no					
